# Different transfer pathways of an organochlorine pesticide across marine tropical food webs assessed with stable isotope analysis

**DOI:** 10.1371/journal.pone.0191335

**Published:** 2018-02-01

**Authors:** Charlotte R. Dromard, Yolande Bouchon-Navaro, Sébastien Cordonnier, Mathilde Guéné, Mireille Harmelin-Vivien, Claude Bouchon

**Affiliations:** 1 UMR BOREA, CNRS 7208 –MNHN–UPMC–UCBN–IRD 207 –UA, Laboratoire d’Excellence « CORAIL », Université des Antilles, Pointe-à-Pitre, Guadeloupe; 2 CNRS/IRD UM 110, Institut Méditerranéen d’Océanologie (MIO), Aix-Marseille Université, Marseille, France; VIT University, INDIA

## Abstract

Chlordecone is a persistent organochlorine pesticide used in the banana fields of the French West Indies from 1972 to 1993. Three marine habitats (mangroves, seagrass beds and coral reefs) of two study sites located downstream contaminated rivers were chosen to evaluate the level of contamination of marine food webs. On each habitat, the food chain collected included suspended organic matter, primary producers (macroalgae, algal turf, seagrass), zooplankton, symbiotic organisms (corals, sea anemones), primary consumers (herbivores, suspension feeders, biofilm feeders), omnivores and detritivores (lobsters, fish), secondary consumers (carnivores 1: invertebrate feeders, planktivores) and tertiary consumers (carnivores 2: invertebrate and fish feeders, piscivores). Log-linear regressions of the concentrations of chlordecone *versus* nitrogen isotopic ratios (δ^15^N) were used to assess the bioaccumulation of chlordecone along trophic food webs. At each site, bioconcentration and bioamplification take part on the transfer of chlordecone in marine organisms. In mangroves (*i*.*e*. close to the source of pollution), lower trophic magnification factors (TMF) indicated that bioconcentration prevailed over bioamplification phenomenon. The opposite phenomenon appeared on coral reefs in which bioconcentration processes were less important and bioamplification pathway became dominant. Far from the source of pollution, molecules of chlordecone seemed to be transfered to organisms mostly *via* trophic interactions rather than water contact.

## Introduction

Persistent organic pollutants (POPs) are organic substances, persistent, bioaccumulative and possess toxic characteristics likely to cause adverse human health or environmental effects. Many thousands of organic trace pollutants, such as organochlorine pesticides (OCPs), polychlorinated biphenyls (PCBs), polybrominated diphenyl ethers (PBDEs) or polycyclic aromatic hydrocarbons (PAHs) are generated by agricultural and industrial activities and are, in part, released into the environment [1,2]. Coastal marine areas are generally the ultimate depositories of these anthropogenic compounds that reach the sea by riverine and atmospheric pathways [3].

In the French West Indies, an organochlorine insecticide called “chlordecone” (commercialized as ®Kepone, then as ®Curlone) was applied to banana fields from 1973 to 1993 to control root borers [4]. This hydrophobic molecule undergoes no significant biotic or abiotic degradation. Consequently, chlordecone is still present in soils where it was applied and the chlordecone pollution of soils in the French West Indies has been estimated to last for decades or centuries [5]. In Guadeloupe, approximately 6 200 hectares are moderately to heavily polluted by chlordecone [6], which represents about 25% of the land surface used for agriculture. Banana plants grow in the southern part of Basse-Terre (one of the two islands of Guadeloupe), which is mountainous and, as a consequence of tropical humid weather, characterized by intense rainfall events. Organochlorine molecules, adsorbed onto organic matter of the soil, end up in the ocean due to runoff and infiltration [5,7,8].

Toxicological studies have demonstrated that chlordecone is a reproductive and developmental toxicant, neurotoxic and carcinogenic for rodents and is an endocrine-disrupting chemical [9,10]. Several surveys have confirmed that French West Indies human population continues to be exposed to this chemical through consumption of contaminated foodstuffs and a correlation between chlordecone exposure and risk of prostate cancer has been evidenced for human [11,12].

Since 2003, several samplings surveys have been conducted in Guadeloupe to evaluate the level of contamination by chlordecone of some species of fish, crustaceans and mollusks, principally species with a high economical interest in order to regulate the fishing activities around the island [13–18]. In 2008, the European Commission of Food Safety set the maximal residue limit (MRL) to 20 μg.kg^-1^ wet weight for the consumption and commercialization of sea products. Research surveys indicated that the most contaminated marine areas are located downstream to banana plantations [16]. These areas are now totally closed to fishing activities and the boundary areas are classified as areas of fishing restrictions in which it is not possible to fish a list of targeted species.

While spatial variations in the level of contamination of marine areas have been identified [15,16], no study has been conducted to understand the contamination dynamics, including the potential ways of transfer and bioaccumulation of the molecule in the food chain.

Persistent hydrophobic chemicals, such as chlordecone, may accumulate in marine organisms through different mechanisms: *via* the direct uptake from surrounding contaminated waters by gills or teguments (bioconcentration) and *via* the consumption of contaminated food (bioamplification or biomagnification) [19]. Bioconcentration is defined as the uptake of a chemical by an organism directly from the abiotic environment, resulting in a concentration of chemical in the organism higher than in the environment [20]. This uptake of chemicals from water probably follows a passive diffusion mechanism analogous to that of oxygen uptake until reaching an equilibrium partitioning between water and biota level [19,21]. Recently, a negative relationship between the level of chlordecone in the lionfish, *Pterois volitans*, and the distance of individuals from the source of pollution has been demonstrated [17]. These results indicated a potential bioconcentration of chlordecone in marine fishes. Bioamplification (or biomagnification) is a contamination by dietary exposure along the food chain. The transfer of chemicals, such as POPs, results in elevated concentrations of the chemical with increasing trophic level, due to their resistance to chemical and metabolic degradations [22].

Biomagnification mechanisms in marine food webs can be evaluated with stable isotope analyses (SIA). Generally, quantitative comparisons are performed using nitrogen stable isotope ratios (δ^15^N) as a proxy for the trophic position of species, because δ^15^N value increases with increasing trophic level [23]. The use of stable isotopes to assess trophic position has numerous advantages over traditional methods such as analysis of gut contents as it averages dietary assimilation over a longer period of time [24]. The regression slope of the concentration of pollutants *versus* δ^15^N signatures of fish has been used to model and predict bioamplification processes [25–27]. At the same time, the y-intercept of the regression line gives an estimation of the level of contamination in the environment, *i*.*e*. the level of the background concentration [25].

While bioconcentration processes received not much attention in the literature, bioamplification of POPs, which include organochlorine pesticides, have been widely studied along food webs of the artic region [28,29], cold seas [30–32], freshwater systems [26,33–36] or temperate marine ecosystems [37–40]. On the contrary, few studies dealt with bioaccumulation processes of POPs on entire trophic food webs in marine tropical ecosystems. In these areas, the level of contamination of organochlorine pollutions has been measured in mangrove food chains [41–45], in vegetal and sediments in seagrass beds [44,46,47] and in macrophytes, invertebrates and fish in coral reefs [44,48–50]. In parallel, few works investigated the contamination of trophic food webs within the continuum “mangrove-seagrass bed-coral reef” [44].

The principal objective of the present study was to evaluate the part of bioconcentration and bioamplification mechanisms in the transfer of chlordecone in marine coastal food webs of Guadeloupe along three interlinked habitats: mangroves, seagrass beds and coral reefs. For that, regression models of the concentrations of chlordecone *versus* nitrogen isotopic ratios were performed on each habitat of two study sites. These models were conducted on the entire food chain from suspended organic matter to piscivorous predators.

## Material and methods

### Study sites

Two study sites (Goyave and Petit-Bourg) were chosen on the eastern coast of Basse-Terre in Guadeloupe. They are located in an area of fishing restrictions due to their position downstream contaminated rivers and banana fields. At each site, three types of marine habitats were investigated: coastal mangroves, seagrass beds (located approximately at 400 m from the coast) and coral reefs (between 500 m and 3 km offshore), representing a seaward gradient of decreasing land influence. Sampling depth was comprised between 1 m in mangroves and 5 m in coral reefs ecosystems.

### Sample collection and preparation

The sampling survey was carried out from January 2014 to February 2015. For this study, 387 samples were collected, 193 at Goyave and 194 at Petit-Bourg (Table 1). The sampling protocol (location of the captures, method of capture and list of the collected species) has been approved and covered by a field permit delivered by the Direction de la Mer of Guadeloupe. The full list of collected species is given in S1 Table. Macroalgae, fishes and crustaceans were collected by hand, spearfishing or using nets in seagrass beds and mangroves. Whenever possible, three replicates were made for each species. To sample the suspended organic matter (SOM), 40 L of seawater were collected in the three habitats at each site in acid-cleaned plastic drums. In order to have three replicates of SOM per habitat at each site, 10 L of seawater were filtered on each Whatman GF/F 47 mm filter. Samples were grouped into eight trophic categories: SOM, primary producers (macroalgae, algal turf, seagrass), zooplankton, symbiotic organisms (corals, sea anemones), primary consumers (herbivores, suspension feeders, biofilm feeders), omnivores and detritivores (lobsters, fish), secondary consumers (carnivores 1: invertebrates feeders, planktivores) and tertiary consumers (carnivores 2: invertebrates and fish feeders, piscivores). Each individual was rinsed, measured (total length, TL, in cm) and weighed (wet weight, ww, in g). For each organism, 50 g of flesh for animal (white mucle) and vegetative tissue were collected and frozen (-18°C) until analyses. Due to the small amount collected, 0.03 g ww of SOM were used for the analyses. Chlordecone concentrations and isotopic ratios were measured on the same sample. Same type (flesh or tissue) and amount of sample was collected at the two sites.

**Table 1:** Number of individuals and species (into brackets) collected in each habitat.

Sites	Goyave			Petit-Bourg		
Habitats	Mangrove	Seagrass	Reef	Mangrove	Seagrass	Reef
SOM	3	3	3	3	3	3
Zooplankton			3			3
Primary producers	6 (2)	15 (5)	12 (4)	6 (2)	15 (5)	11 (4)
Symbiotic organisms		3 (1)	8 (3)			9 (3)
Primary consumers	6 (2)	16 (6)	19 (7)	3 (1)	22 (9)	12 (6)
Detritivores—omnivores	9 (5)	4 (2)	9 (3)	14 (6)	3 (1)	5 (2)
Secondary consumers	23 (11)	14 (7)	9 (4)	18 (8)	13 (6)	15 (5)
Tertiary consumers	12 (10)	4 (2)	12 (5)	17 (8)	7 (3)	12 (5)
**Total**	**59 (30)**	**59 (23)**	**75 (26)**	**61 (25)**	**63 (24)**	**70 (25)**

SOM: suspended organic matter. The full list of species is given in S1 Table.

### Chlordecone extraction and analysis

Chlordecone was extracted from homogenized sampled tissues with a solution of organic solvents (hexane-acetone) and turned into chlordecone hydrate (hydrosoluble) in the presence of sodium hydroxide. The aqueous phase was rinsed with hexane to eliminate fats. Chlordecone was then reassembled in acid conditions, extracted with a solution of hexane and acetone. Concentration of chlordecone was quantified with liquid chromatography coupled to mass spectrometry in tandem (UPLC-MS/MS). Chlordecone concentrations were expressed in μg.kg^-1^ (ww) and the lower limit of quantification (LOQ) with this method was 1 μg.kg^-1^ (ww) with measurement precision of 0.1 μg.kg^-1^ when data were superior to the LOQ. In this study, chlordecone concentration was not lipid-corrected as the maximal residue limit indicated by European regulations is expressed in wet weight.

### Stable isotope analyses

All samples were cut into small pieces, oven dried at 50°C to a constant weight and ground into a homogenous fine powder. For each sample, 1 mg of dry weight (dw) was used for analysis. Nitrogen isotope ratios were determined by a continuous flow mass spectrometer (Thermo Fisher™, delta V Advantage). Nitrogen isotope ratios were expressed in standard delta notation (δ^15^N) in ‰ according to the following formula: δ^15^N = [(R_sample_/R_standard_− 1)] x 1000, where R is the ratio ^15^N:^14^N of samples or international standard (atmospheric air). The analytical measurement precision was <0.1‰ (replicate measurements of internal laboratory standards).

### Trophic magnification factor (TMF)

The trophic transfer of chlordecone through the food chain is based on the relationship between nitrogen isotope ratios (δ^15^N in ‰) and chlordecone concentrations ([CHD] in μg.kg^-1^). The trophic transfer of chlordecone was estimated following the formula [32]: log_10_[CHD] = a δ^15^N + b, where *b* is the y-intercept (constant dependent on the background concentration) and *a* is the slope of the regression log_10_[CHD] function to δ^15^N (indicating the biomagnification power of the contaminant).

The trophic magnification factor (TMF), also called “food web magnification factor” (FWMF), is calculated from the slope using the following formula: TMF = e^a^.

We replace trophic level (TL) (used in [32]) by δ^15^N values as a more accurate estimation of the organism position in the food chain. Contaminant with TMF greater than 1 is considered to biomagnify in the food chain while, TMF values comprised between 0 and 1 indicates that the contaminant is not biomagnified in the food web. A TMF value inferior to zero indicates that the contaminant is excreted by the organisms in the food chain [25,45].

### Statistical analysis

Normality of data was verified with Shapiro-Wilk tests. Concentrations of chlordecone were compared between habitats (mangrove, seagrass beds and reef) with one-way analyses of variance (ANOVA), followed by multiple comparison tests, performed with post hoc Tukey’s honestly significant difference (HSD) test. Simple linear regression analyses were used to investigate the relationship between the logarithm of chlordecone concentrations (log_10_[CHD]) and the trophic level of species (δ^15^N). Comparisons of the regression slopes at each site were performed with Student’s t-tests.

Differences of chlordecone concentrations between Goyave and Petit-Bourg were tested with one-way analyses of variance on ranks (Kruskal-Wallis tests) on species present at both sites in the same habitat. To avoid an influence of fish size on between-site comparisons, differences of the mean total length of fish between sites were previously tested with Kruskal-Wallis tests, as the influence of fish size on their level of contamination has already been demonstrated [17,39].

## Results

### Levels of contamination by chlordecone according to habitat

In Goyave, the concentrations of chlordecone varied from 1.1 μg.kg^-1^ for *Halimeda incrassata* to 1034 μg.kg^-1^ for an individual of *Pomadasys corvinaeformis*. At this site, concentrations of chlordecone were significantly different according to habitat (ANOVA, F_(2,190)_ = 26.9, p < 0.0001). The highest mean concentration of chlordecone (± standard error) was found in mangrove (182.4 ± 22.6 μg.kg^-1^). This mean value was significantly higher from those measured in seagrass beds and coral reef (Tukey’s HSD, p < 0.0001). Mean concentration of chlordecone in seagrass bed (54.1 ± 8.9 μg.kg^-1^) and coral reef (61.1 ± 8.4 μg.kg^-1^) did not differ significantly (Tukey’s HSD, p = 0.94). In mangrove, only 6 samples (10% of mangrove samples) presented a concentration lower to the MRL (= 20 μg.kg^-1^). The number of samples compliant with the MRL was 28 in seagrass beds (47%) and 37 in coral reefs (45%).

**Fig 1:**
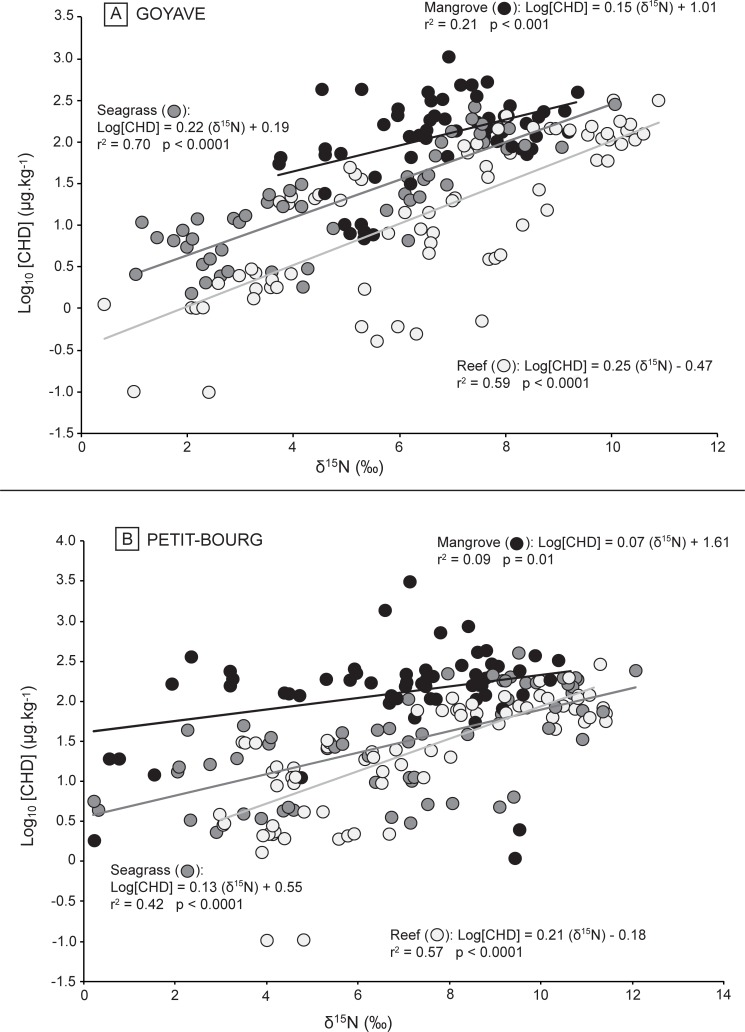
Relationships between logarithm of chlordecone concentrations (log_10_[CHD]) and nitrogen isotope ratios (δ^15^N). Organisms measured in mangrove (black circle), seagrass beds (grey circles) and coral reefs (white circles) at A) Goyave and B) Petit-Bourg.

In Petit-Bourg, concentrations of chlordecone varied from 1.1 μg.kg^-1^ for *Megalops atlanticus* to 3012 μg.kg^-1^ for an individual *Callinectes* sp. Similarly to Goyave, the mean concentration of chlordecone measured in mangrove was the highest (251.0 ± 52.7 μg.kg^-1^) and was significantly different from those of the two other habitats (ANOVA, F_(2,191)_ = 13.4, p < 0.0001). No significant difference was found between the concentrations measured in seagrass (71.5 ± 10.6 μg.kg^-1^) and coral reef communities (48.5 ± 6.0 μg.kg^-1^) (Tukey’s HSD, p < 0.85). The number of samples with a concentration of chlordecone lower to the MRL was 8 in mangrove (13% of mangrove samples), 23 in seagrass beds (36%) and 32 in coral reefs (45%). The level of contamination in each habitat was also reflected by the y-intercept (*b*) of simple linear regressions log_10_[CHD] *versus* δ^15^N of organisms (Fig 1). The values of the constant *b* differed significantly with habitat, showing a consistent pattern. At the two sites, the y-intercept was the highest in mangroves (*b* = 1.01 in Goyave and *b* = 1.61 in Petit-Bourg), intermediate in seagrass beds (*b* = 0.19 in Goyave and *b* = 0.55 in Petit-Bourg), and the lowest in coral reefs (*b* = -0.12 in Goyave and *b* = 0.01 in Petit-Bourg).

### Bioamplification of chlordecone along marine food webs

A significant relationship was found between log-transformed concentrations of chlordecone (log_10_[CHD]) *versus* δ^15^N signatures of organisms in each habitat in Goyave (mangrove: r^2^ = 0.21, p < 0.01, seagrass bed: r^2^ = 0.70, p < 0.0001, coral reef: r^2^ = 0.64, p < 0.0001) (Fig 1A) and in Petit-Bourg (mangrove: r^2^ = 0.09, p < 0.01, seagrass bed: r^2^ = 0.42, p < 0.0001, coral reef: r^2^ = 0.63, p < 0.0001) (Fig 1B).

Trophic magnification factors (TMF) were calculated based on the slope of each model of linear regression. In Goyave, TMF was higher than 1 in mangrove (TMF_mangrove_ = 1.16), seagrass bed (TMF_seagrass_ = 1.25) and coral reef (TMF_reef_ = 1.25). These values were also higher than 1 in the second site Petit-Bourg (TMF_mangrove_ = 1.07, TMF_seagrass_ = 1.14, TMF_reef_ = 1.21). At each site, increasing TMF values were found across a seaward spatial gradient from mangroves to coral reefs.

At Goyave, slopes of the linear regressions were significantly different between mangrove and seagrass beds (Student’s t-test, p<0.05) and between mangrove and coral reef systems (Student’s t-test, p<0.05), but were not different between seagrass beds and coral reef (Student’s t-test, p>0.05).

At Petit-Bourg, slopes were not different between mangrove and seagrass beds (Student’s t-test, p>0.05), but the slopes of these two systems differed significantly with those of coral reef (Student’s t-tests, p<0.05).

### Difference of contamination between sites

Differences of chlordecone concentrations between Goyave and Petit-Bourg were tested on a list of species present at both sites in the same habitat (Table 2). *Aulostomus maculatus*, *Haemulon carbonarium* and *Lutjanus apodus* were present in coral reefs at both sites, but were not considered for site comparisons because total of individuals differed significantly with site (Kruskal-Wallis, p<0.05 for each considered species). In mangrove, 57% (4/7) of the studied species showed a concentration of chlordecone higher at Petit-Bourg than at Goyave. In seagrass beds, 30% (3/10) of the studied species followed a similar tendency. Finally, higher concentrations of chlordecone were measured at Petit-Bourg for 20% (2/10) of samples collected in coral reefs. Two species, the sea anemone *Stichodactyla helianthus* and the West indian starsnail *Lithopoma tectum*, presented higher chlordecone concentrations at Goyave than Petit-Bourg (Table 2). The other species did not show any significant difference in their concentrations of chlordecone between Petit-Bourg and Goyave.

**Table 2:** Comparisons of concentrations of chlordecone between the two sites.

		Petit-Bourg	Goyave			
Mangrove	*n*	[CHD]	[CHD]	X^2^	p values	Comparisons
SOM	6	191.3 ± 38.5	60.0 ± 5.6	3.86	p < 0.05	PB > G
*Acanthophora spicifera*	6	11.3 ± 0.6	7.6 ± 0.6	3.97	p < 0.05	PB > G
*Anchoa lyolepis*	6	323.7 ± 47.5	209.0 ± 101.9	2.33	p = 0.13	PB = G
*Callinectes* sp.	6	1547.3 ± 1387.8	257.0 ± 52.1	2.33	p = 0.13	PB = G
*Crassostrea rhizophorae*	6	122.3 ± 3.8	74.7 ± 5.5	3.86	p < 0.05	PB > G
*Eucinostomus gula*	6	202.3 ± 12.9	100.7 ± 14.6	3.87	p < 0.05	PB > G
*Harengula clupeola*	5	265.0 ± 137.2	113.0 ± 72.5	1.33	p = 0.25	PB = G
**Seagrass beds**						
SOM	6	31.7 ± 2.9	27.3 ± 9.5	0.44	P = 0.5	PB = G
*Caulerpa sertularoides*	6	16.6 ± 6.0	10.7 ± 2.0	1.19	P = 0.4	PB = G
*Cerithium vulgatum*	6	27.0 ± 1.0	22.7 ± 4.9	3.14	P = 0.07	PB = G
*Halophila stipulacea*	6	4.6 ± 0.9	4.1 ± 0.8	0.43	P = 0.70	PB = G
*Holothuria Mexicana*	6	3.9 ± 1.1	4.1 ± 2.1	0.20	P = 0.66	PB = G
*Neopetrosia carbonaria*	6	14.7 ± 1.5	8.8 ± 3.3	3.86	P < 0.05	PB > G
*Padina* sp.	6	4.5 ± 0.3	1.8 ± 0.2	3.86	P < 0.05	PB > G
*Sparisoma radians*	6	63.3 ± 37.2	19.0 ± 3.6	3.86	P < 0.05	PB > G
*Syringodium filiforme*	6	5.4 ± 0.8	6.9 ± 0.3	3.86	P = 0.10	PB = G
*Thalassia testudinum*	6	3.0 ± 0.6	2.7 ± 0.2	0.43	P = 0.70	PB = G
**Coral reefs**						
SOM	6	30.3 ± 2.1	20.7 ± 0.6	3.97	P < 0.05	PB > G
Plankton	6	20.7 ± 2.1	6.3 ± 1.7	3.86	P < 0.05	PB > G
*Halimeda incrassata*	5	1.8 ± 1.1	1.3 ± 1.3	3.00	P = 0.08	PB = G
*Lithopoma tectum*	6	13.0 ± 2.0	21.3 ± 1.5	3.86	P < 0.05	PB < G
*Panulirus argus*	6	86.7 ± 18.5	86.7 ± 10.4	0.05	P = 0.83	PB = G
*Porites astreoides*	5	2.4 ± 0.5	1.6 ± 0.4	3.00	P = 0.08	PB = G
*Porites furcata*	6	1.9 ± 0.5	2.6 ± 0.4	3.14	P = 0.07	PB = G
*Pterois volitans*	6	74.3 ± 11.7	87.7 ± 26.1	0.43	P = 0.50	PB = G
*Scarus taeniopterus*	6	11.2 ± 1.7	10.3 ± 3.2	0.43	P = 0.51	PB = G
*Stichodactyla helianthus*	6	11.5 ± 2.3	41.7 ± 6.0	3.86	P < 0.05	PB < G

Comparisons were done with Kruskal Wallis tests. [CHD]: concentrations of chlordecone (± SE in μg.kg^-1^), SOM: suspended organic matter, PB: Petit-Bourg, G: Goyave, *n* is the number of samples.

## Discussion

The highest concentrations of chlordecone were measured in organisms collected in mangroves that are located on the coastline, close to river mouths. The level of chlordecone was three to four times lower in the organisms living in seagrass beds and coral reefs, two habitats located between 400 m and 3 km from the coast respectively.

This decreasing gradient of chlordecone from the coast to the open sea was previously identified [18] when studying spatial variations of the level of chlordecone contamination in different trophic groups. These results indicated that marine organisms are contaminated by chlordecone by direct contact with seawater and highlighted the existence of a bioconcentration phenomenon in these areas. Through water contact, living organisms integrate organochlorine toxicant *via* gills and tegument epithelium, and their level of residue is related to the level of contamination the ambient water [1]. The measurement of chlordecone concentration directly in seawater was not possible in the present study due to analytical issues. Therefore, it was not possible to calculate the concentration factor (relation between the contaminant concentration of organisms and those of the ambient water) in these food webs. However, the y-intercept of the regression lines (log_10_[CHD] *vs* δ^15^N), the constant *b*, gave a relative estimation of the ambient level of chlordecone in each habitat. At both sites, the constant *b* was maximal in mangroves, intermediate in seagrass beds and minimal in coral reefs, attesting to the existence of a decreasing gradient of contamination seawards from the coast. These results indicated that the mechanism of bioconcentration is prevailing in mangroves, inducing a contamination of the entire food chain, with less influence of the trophic level of the organisms. In mangroves, a large part of samples (53/59 samples) presented contamination values higher than the MRL authorized for consumption and commercialization of sea products in these areas. Bioconcentration seemed to be a less important driver of contamination by chlordecone in the two other habitats.

At each site, the logarithms of chlordecone concentrations were positively correlated to the trophic level of marine organisms estimated by their δ^15^N. These observations indicated that the concentration of contaminant increased along the food chain, from lower to higher trophic levels, attesting of the existence of a bioamplification phenomenon in each habitat. This mode of transfer occurs through food consumption and the integration of pollutant *via* digestive pathways. Bioamplification of POPs is thus related to the feeding habits and trophic level of organisms [26,51]. However, it should be noted that the variability of chlordecone concentrations among individuals in one species and among species at each trophic level was much higher than the variability of their δ^15^N (see S1 Table). Such a high intra- and inter-specific variability of contaminant concentration is observed in all studies, whatever the type of contaminants [39], as the level of contamination in one organism depends on many factors, such as the environmental conditions (temperature, salinity habitat, contaminant inputs,), and the biology (species, diet, trophic level) and physiology (metabolism, growth rate, sex, age) of the different organisms studied ([36,32] among many others). To fully apprehend the transfer of any contaminant in food webs it is necessary to analyze a sufficiently wide spectrum of species and individuals.

Bioamplification, increasing contaminant concentration with increasing trophic level, was demonstrated by the measurement of Trophic Magnification Factors (TMF), based on the slopes of the regression lines (log_10_[CHD] *vs* δ^15^N). A contaminant presenting a TMF higher than 1 is considered to biomagnify in the food chain, while a TMF value comprised between 0 and 1 indicates that the contaminant is not biomagnified [25,45]. In the present study, all TMF values exceeded 1, indicating that the level of chlordecone bioamplify along the food webs. These TMF values ranged from 1.07 to 1.25, and reached similar thresholds than those calculated for POPs in entire food webs (from zooplankton to apex predators) in African lake (TMF_DDT_ = 1.16, TMF_DDE_ = 1.25, [26]). On the contrary, the TMF values of the present study were lower than those calculated in South China mangroves (TMF_DDT_ = 2.61, TMF_PCB_ = 2.76, [45]), in Artic food webs exposed to PCB and DDT (TMF = 2.41 and 2.20 respectively, [37]), or exposed to Mirex pollution (TMF = 10.5, [32]). However, the length of the food chain greatly influences the value of biomagnification factors. Studies conducted in the Artic or cold sea regions generally encompass a larger scale of trophic levels (from primary producers to marine mammals and sea birds) than in other ecosystems.

In the present study, TMF values were different according to the habitat, with minimal values in mangroves, intermediate values in seagrass beds (at Petit Bourg) and maximal values in coral reefs. The statistical comparisons of the regression slopes indicated that degrees of bioamplification were different according to the three habitats. At Goyave, the highest slopes were found in seagrass beds and coral reefs and these values differed significantly from the slope calculated in mangrove. These results indicate that, at this site, bioamplification is maximal in seagrass beds and coral reefs food webs and minimal in mangrove systems. At Petit Bourg, the highest slope was found in coral reef ecosystem and differed from those calculated in mangrove and seagrass beds. At this site, bioamplification seemed to be maximal in coral reef, but lower and equivalent in seagrass beds and mangrove. In Singapore, higher POPs concentrations were found in predator species while organisms at lower trophic levels had in general, lower levels of POPs [43]. These results highlighted a bioamplification phenomenon in mangrove food webs in Singapore. In the present study, the difference of TMF values and the comparisons of the regression slopes between the three habitats showed that the bioamplification phenomenon is masked in mangroves by the predominance of bioconcentration. This tendency also appears in seagrass beds at Petit-Bourg, which can be explained by the difference of background concentrations between the two sites. At petit-Bourg, the level of contamination in the environment seems to be higher than at Goyave, leading to a higher influence of the source of pollution on the food web and a prevalence of bioconcentration phenomenon as far as seagrass beds.

Biomagnification is linked to the physical properties of contaminant such as the octanol-water partition coefficient (log *K*_OW_). Concentrations of very hydrophobic substances (log *K*_OW_ > 6.3) increased with trophic positions, attesting that bioamplification occurred for these molecules [36]. For less hydrophobic substances of log *K*_OW_ < 5.5, biomagnification is not observed and the contamination of the food chain is the result of equilibrium partition of the chemical between water and biota level. Our colleagues [52] predicted that compounds with log *K*_OW_ < 6, such as chlordecone (*K*_OW_ = 5.41), attain equilibrium within 1 year. Some evidence for biomagnification of chemical with log *K*_OW_ values between 5.5 and 6.3 has been demonstrated [36]. Moreover, the ability of Kepone to magnify, like in the present study, while the *K*_OW_ of chlordecone is less than 5.5, has been evidenced before [53,54].

The level of contamination, respectively to each habitat, appears to be globally similar between the two sites (Goyave and Petit-Bourg). However, between 20 and 57% of the samples, depending on the habitat, showed higher concentrations of chlordecone in Petit-Bourg than in Goyave. The level of contamination of rivers cannot explain this result, because the river flowing at Goyave is more contaminated than these flowing at Petit-Bourg (“Rivière Moustique” and “Rivière la Rose”) (Office de l’Eau Guadeloupe). However, the location of the two sites could give an explanation to this difference in the level of contamination. Petit-Bourg is located in the North of Goyave and is more enclosed in the bay of Petit Cul-de-Sac Marin, where the trade winds tend to accumulate seawater. This particularity could involve a trapping of the molecules of chlordecone in the north of the bay.

In summary, both phenomena (bioconcentration and bioamplification) are effective on the transfer of chlordecone in marine organisms in Guadeloupe, but bioconcentration prevails on bioamplification in mangroves at both sites, and in seagrass beds at Petit-Bourg. In coral reefs at both sites and seagrass beds in Goyave, the opposite tendency seems to appear. Far from the source of pollution, molecules of chlordecone were transferred more *via* trophic interactions than water contact.

Consequences of chlordecone exposure to marine organisms have not been widely studied while the knowledge on their damages on human health is well documented [11,12,54]. However, few studies were conducted to investigate the consequences of long-term exposure of marine food chain or the metabolic and physiologic responses of marine organisms facing with chlordecone exposure [55].

## Supporting information

S1 Table List of the species collected in each habitat at Goyave and Petit-Bourg. [CHD]: mean concentration of chlordecone (± SE in μg.kg^-1^), δ^15^N: nitrogen isotope ratios (in ‰). *n* is the number of sample. T: Teleostei, E: Echidermata, P: Porifera; M: Mollusca, C: Crustacea, A: Annelida, Cn: Cnidaria.
(DOC)
