# Supplementary material for: Different transfer pathways of an organochlorine pesticide across marine tropical food webs assessed with stable isotope analysis
Source: PLoS One. 2018 Feb 1;13(2):e0191335. doi: 10.1371/journal.pone.0191335 (PMC5794063; doi:10.1371/journal.pone.0191335)
Supplement: S1 Table — [CHD]: mean concentration of chlordecone (± SE in μg.kg-1), δ15N: nitrogen isotope ratios (in ‰). n is the number of sample. T: Teleostei, E: Echidermata, P: Porifera; M: Mollusca, C: Crustacea, A: Annelida, Cn: Cnidaria. (DOC) [file pone.0191335.s001.doc]

| **Sites** |  |  | **Goyave** | | | | | | **Petit-Bourg** | | | | | |
| --- | --- | --- | --- | --- | --- | --- | --- | --- | --- | --- | --- | --- | --- | --- |
| **Habitats** |  |  | **Mangrove** | | **Seagrass bed** | | **Coral reef** | | **Mangrove** | | **Seagrass bed** | | **Coral reef** | |
|  |  | n | [CHD] | δ^15^N | [CHD] | δ^15^N | [CHD] | δ^15^N | [CHD] | δ^15^N | [CHD] | δ^15^N | [CHD] | δ^15^N |
| SOM |  | 18 | 60.0 ± 5.6 | 3.7 ± 0.0 | 27.3 ± 9.5 | 6.2 ± 0.0 | 20.7 ± 0.6 | 7.0 ± 0.0 | 191.3 ± 38.5 | 3.2 ± 0.0 | 31.7 ± 2.9 | 4.1 ± 0.0 | 30.3 ± 2.1 | 5.3 ± 0.0 |
| Zooplankton |  | 6 |  |  |  |  | 6.3 ± 1.7 | 6.6 ± 0.0 |  |  |  |  | 20.7 ± 2.1 | 6.3 ± 0.1 |
| **Primary producers** |  |  |  |  |  |  |  |  |  |  |  |  |  |  |
| Algal turf |  | 5 |  |  |  |  | < 1 | 2.2 ± 0.1 |  |  |  |  | 4.1 | 5.0 |
| *Acanthophora spicifera* |  | 9 | 7.6 ± 0.6 | 5.3 ± 0.2 |  |  | 2.3 | 2.7 | 11.3 ± 0.6 | 3.7 ± 1.8 |  |  |  |  |
| *Caulerpa sertularoides* |  | 9 |  |  | 10.7 ± 2.0 | 4.3 ± 1.0 |  |  |  |  | 16.6 ± 6.0 | 5.3 ± 1.7 | 30.3 ± 0.6 | 3.6 ± 0.1 |
| *Ulva flexuosa* |  | 3 | 9.6 ± 0.8 | 5.2 ± 0.2 |  |  |  |  |  |  |  |  |  |  |
| *Galaxaura rugosa* |  | 6 |  |  |  |  | 2.1 ± 0.6 | 3.4 ± 0.2 |  |  |  |  | 2.2 | 4.3 ± 0.4 |
| *Halimeda incrassata* |  | 6 |  |  |  |  | 1.9 | 0.8 |  |  |  |  | 3.2 ± 0.5 | 3.0 ± 0.1 |
| *Padina* sp. |  | 6 |  |  | 1.8 ± 0.3 | 2.9 ± 1.2 |  |  |  |  | 4.5 ± 0.3 | 4.5 ± 0.1 |  |  |
| *Halophila stipulacea* |  | 9 |  |  | 4.1 ± 0.8 | 2.5 ± 0.2 |  |  | 13.3 ± 9.9 |  | 4.6 ± 0.9 | 1.3 ± 1.9 |  |  |
| *Syringodium filiforme* |  | 6 |  |  | 6.9 ± 0.3 | 1.8 ± 0.3 |  |  |  |  | 5.4 ± 0.8 | 8.9 ± 0.7 |  |  |
| *Thalassia testudinum* |  | 6 |  |  | 2.7 ± 0.2 | 2.9 ± 0.3 |  |  |  |  | 3.0 ± 0.6 | 3.0 ± 0.8 |  |  |
| **Primary consumers** |  |  |  |  |  |  |  |  |  |  |  |  |  |  |
| *Acanthurus bahianus* | T | 2 |  |  |  |  |  |  |  |  | 50.5 ± 27.6 | 8.1 ± 1.4 |  |  |
| *Amphimedon compressa* | P | 6 |  |  |  |  | 4.1 | 7.8 ± 0.1 |  |  | 10.7 ± 0.6 | 7.2 ± 0.1 |  |  |
| *Cerithium vulgatum* | M | 6 |  |  | 22.7 ± 4.9 | 4.0 ± 0.1 |  |  |  |  | 27.0 ± 1.0 | 5.4 ± 0.1 |  |  |
| *Crassostrea rhizophorae* | M | 6 | 74.7 ± 5.5 | 4.7 ± 0.2 |  |  |  |  | 122.3 ± 3.8 | 4.5 ± 0.2 |  |  |  |  |
| *Holothuria mexicana* | E | 10 |  |  | 4.1 ± 2.1 | 4.7 ± 1.3 | 1.7 | 6.4 ± 1.1 |  |  | 3.9 ± 1.1 | 7.1 ± 0.4 | 1.9 | 5.6 |
| *Lithopoma tectum* | M | 6 |  |  |  |  | 21.3 ± 1.5 | 4.6 ± 0.3 |  |  |  |  | 13.0 ± 2.0 | 4.3 ± 0.2 |
| *Mithrax spinosissimus* | C | 1 |  |  |  |  | 14.0 | 6.6 |  |  |  |  |  |  |
| *Neopetrosia carbonaria* | P | 6 |  |  | 8.8 ± 3.3 | 2.0 ± 0.1 |  |  |  |  | 14.7 ± 1.5 | 2.3 ± 0.4 |  |  |
| *Pinna carnea* | M | 2 |  |  | 31.0 | 4.2 |  |  |  |  | 44.0 | 6.4 |  |  |
| *Sabellastarte magnifica* | A | 6 |  |  |  |  | < 1 | 5.6 ± 0.3 |  |  |  |  | 2.2 ± 0.1 | 6.1 ± 0.5 |
| *Scarus taeniopterus* | T | 6 |  |  |  |  | 10.3 ± 3.2 | 6.1 ± 0.3 |  |  |  |  | 11.2 ± 1.7 | 6.8 ± 0.5 |
| *Sparisoma radians* | T | 9 | 42.0 ± 20.3 | 5.4 ± 0.8 | 19.0 ± 3.6 | 6.4 ± 0.6 |  |  |  |  | 63.3 ± 37.2 | 7.4 ± 0.9 |  |  |
| *Sparisoma rubripinne* | T | 1 |  |  |  |  |  |  |  |  |  |  | 24.0 | 7.6 |
| *Lobatus costatus* | M | 3 |  |  |  |  |  |  |  |  | 37.3 ± 7.4 | 4.5 ± 2.0 |  |  |
| *Lobatus gigas* | M | 8 |  |  | 19.7 ± 3.1 | 3.6 ± 0.2 | 20.0 ± 1.7 | 3.9 ± 0.1 |  |  | 50.0 | 3.5 | 8.7 | 4.2 |
| **Secondary consumers** |  |  |  |  |  |  |  |  |  |  |  |  |  |  |
| *Anchoa lyolepis* | T | 6 | 209.0 ± 101.9 | 7.7 ± 0.8 |  |  |  |  | 323.7 ± 47.5 | 9.8 ± 0.7 |  |  |  |  |
| *Chaetodon capistratus* | T | 2 |  |  |  |  |  |  |  |  | 196.0 | 9.9 |  |  |
| *Eucinostomus argenteus* | T | 3 |  |  |  |  |  |  |  |  | 80.7 ± 46.1 | 10.6 ± 0.3 |  |  |
| *Eucinostomus gula* | T | 9 | 100.7 ± 14.6 | 8.0 ± 0.4 |  |  |  |  | 202.3 ± 12.9 | 8.7 ± 0.2 | 75.7 ± 30.0 | 10.3 ± 1.0 |  |  |
| *Eucinostomus lefroyi* | T | 3 |  |  | 91.3 ± 11.0 | 8.3 ± 0.7 |  |  |  |  |  |  |  |  |
| *Eugerres brasiliensis* | T | 1 |  |  |  |  |  |  | 861.0 | 8.4 |  |  |  |  |
| *Gerres cinereus* | T | 4 | 207.0 | 6.7 |  |  |  |  | 182.5 ± 145.0 | 8.7 ± 0.6 | 76.0 | 10.9 |  |  |
| *Haemulon carbonarium* | T | 7 |  |  |  |  | 204.3 ± 95.7 | 10.3 ± 0.3 |  |  |  |  | 89.5 ± 33.9 | 10.8 ± 0.4 |
| *Haemulon flavolineatum* | T | 3 |  |  |  |  |  |  |  |  |  |  | 66.3 ± 12.3 | 10.9 ± 0.2 |
| *Haemulon plumieri* | T | 3 |  |  |  |  | 112.3 ± 48.9 | 9.7 ± 0.2 |  |  |  |  |  |  |

| **Sites** |  |  | **Goyave** | | | | | | **Petit-Bourg** | | | | | |
| --- | --- | --- | --- | --- | --- | --- | --- | --- | --- | --- | --- | --- | --- | --- |
| **Habitats** |  |  | **Mangrove** | | **Seagrass bed** | | **Coral reef** | | **Mangrove** | | **Seagrass bed** | | **Coral reef** | |
|  |  | n | [CHD] | δ^15^N | [CHD] | δ^15^N | [CHD] | δ^15^N | [CHD] | δ^15^N | [CHD] | δ^15^N | [CHD] | δ^15^N |
| *Halichoeres bivittatus* | T | 2 |  |  | 87.5 | 8.2 |  |  |  |  |  |  |  |  |
| *Halichoeres radiatus* | T | 2 |  |  |  |  |  |  |  |  |  |  | 188.5 ± 10.6 | 10.3 ± 0.4 |
| *Harengula clupeola* | T | 5 | 113.0 ± 72.5 | 6.9 ± 0.6 |  |  |  |  | 265.0 | 5.5 |  |  |  |  |
| *Hemiramphus balao* | T | 4 | 228.5 | 6.7 | 129.0 | 7.4 |  |  |  |  |  |  |  |  |
| *Heteropriacanthus cruentatus* | T | 2 |  |  |  |  | 44.0 | 7.7 |  |  |  |  |  |  |
| *Larimus breviceps* | T | 1 | 522.0 | 7.7 |  |  |  |  |  |  |  |  |  |  |
| *Mulloidichthys martinicus* | T | 1 | 204.0 | 8.0 |  |  |  |  |  |  |  |  |  |  |
| *Myripristis jacobus* | T | 5 |  |  | 206.0 | 8.0 | 209.0 ± 2.1 | 8.0 |  |  |  |  | 104.3 ± 19.5 | 9.6 ± |
| *Octopus vulgaris* | M | 7 |  |  | 35.7 ± 4.5 | 6.6 ± 0.2 |  |  |  |  | 39.0 | 7.4 | 20.3 ± 4.5 | 7.3 ± 0.7 |
| *Ocyurus chrysurus* | T | 4 |  |  | 145.0 | 8.1 |  |  |  |  | 171.7 ± 21.1 | 10.7 ± 0.1 |  |  |
| *Polydactylus virginicus* | T | 2 | 215.5 | 8.7 |  |  |  |  |  |  |  |  |  |  |
| *Pomadasys corvinaeformis* | T | 4 | 524.3 ± 458.9 | 8.0 ± 1.2 |  |  |  |  | 121.0 | 8.8 |  |  |  |  |
| *Sphoeroides greeleyi* | T | 5 | 254.3 ± 196.6 | 6.5 ± 0.8 | 132.0 ± 43.8 | 7.2 ± 0.5 |  |  |  |  |  |  |  |  |
| *Sphoeroides testudinum* | T | 3 |  |  |  |  |  |  | 519.0 ± 168.4 | 8.4 ± 0.5 |  |  |  |  |
| *Trachinotus falcatus* | T | 4 | 429.0 | 5.3 |  |  |  |  | 131.3 ± 21.4 | 7.9 ± 1.5 |  |  |  |  |
| **Tertiary consumers** |  |  |  |  |  |  |  |  |  |  |  |  |  |  |
| *Aulostomus maculatus* | T | 6 |  |  |  |  | 118.0 ± 39.1 | 8.7 ± 0.6 |  |  |  |  | 83.0 ± 7.8 | 8.3 ± 0.2 |
| *Bairdiella ronchus* | T | 3 | 110.0 ± 24.8 | 8.7 ± 0.5 |  |  |  |  |  |  |  |  |  |  |
| *Carangoides bartholomaei* | T | 3 |  |  |  |  |  |  | 173.0 ± 10.4 | 6.5 ± 0.8 |  |  |  |  |
| *Caranx crysos* | T | 8 |  |  | 154.0 ± 32.2 | 7.6 ± 0.2 |  |  | 173.0 ± 77.0 | 7.1 ± 0.4 |  |  | 81.0 | 9.6 |
| *Caranx latus* | T | 5 | 480.0 | 7.2 |  |  |  |  | 180.3 ± 81.3 | 7.9 ± 1.1 |  |  |  |  |
| *Dasyatis centroura* | T | 1 | 88.0 | 8.1 |  |  |  |  |  |  |  |  |  |  |
| *Gymnothorax funebris* | T | 1 | 234.0 | 9.1 |  |  |  |  |  |  |  |  |  |  |
| *Gymnothorax vicinus* | T | 1 | 70.0 | 8.4 |  |  |  |  |  |  |  |  |  |  |
| *Lutjanus apodus* | T | 10 | 180.0 | 6.6 |  |  | 123.7 ± 32.3 | 10.3 ± 0.3 |  |  | 196.3 ± 28.0 | 9.1 ± 1.0 | 160.3 | 11.3 |
| *Lutjanus griseus* | T | 5 |  |  | 284.0 | 10.1 |  |  | 153.0 | 8.7 | 210.3 ± 27.4 | 11.1 ± 0.9 |  |  |
| *Lutjanus mahogani* | T | 1 | 275.0 | 8.1 |  |  |  |  |  |  |  |  |  |  |
| *Lutjanus synagris* | T | 3 | 146.0 | 7.7 |  |  | 130.5 | 10.1 |  |  |  |  |  |  |
| *Megalops atlanticus* | T | 2 |  |  |  |  |  |  | 1.8 | 9.5 |  |  |  |  |
| *Pterois volitans* | T | 6 |  |  |  |  | 87.7 ± 26.1 | 9.9 ± 0.2 |  |  |  |  | 74.3 ± 11.7 | 10.0 ± 0.7 |
| *Rypticus saponaceus* | T | 2 | 207.0 | 8.1 |  |  |  |  | 166.0 ± 1.9 | 1.9 |  |  |  |  |
| *Sphyraena barracuda* | T | 4 |  |  |  |  | 318.0 | 10.9 | 278.0 | 8.9 | 169.0 | 9.7 | 57.0 | 11.4 |
| *Sphyraena picudilla* | T | 1 | 195.0 | 7.7 |  |  |  |  |  |  |  |  |  |  |
| *Tylosurus crocodilus* | T | 2 |  |  |  |  |  |  | 188.0 | 10.4 |  |  |  |  |
| **Detritivores - Omnivores** |  |  |  |  |  |  |  |  |  |  |  |  |  |  |
| *Archosargus rhomboidalis* | T | 3 |  |  |  |  |  |  | 105.7 ± 50.5 | 8.5 ± 0.0 |  |  |  |  |
| *Callinectes* sp. | C | 6 | 257.0 ± 52.1 | 6.2 ± 0.4 |  |  |  |  | 1547.3 ± 1387.8 | 6.6 ± 0.6 |  |  |  |  |
| *Diapterus rhombeus* | T | 4 | 137.0 | 6.5 |  |  |  |  | 136.7 ± 51.4 | 7.6 ± 0.1 |  |  |  |  |
| *Hyporhamphus unifasciatus* | T | 4 | 122.0 | 6.5 |  |  |  |  | 123.7 ± 27.2 | 7.2 ± 0.4 |  |  |  |  |
| *Mugil curema* | T | 3 | 220.0 ± 123.8 | 6.8 ± 0.6 |  |  |  |  |  |  |  |  |  |  |

| **Sites** |  |  | **Goyave** | | | | | | **Petit-Bourg** | | | | | |
| --- | --- | --- | --- | --- | --- | --- | --- | --- | --- | --- | --- | --- | --- | --- |
| **Habitats** |  |  | **Mangrove** | | **Seagrass bed** | | **Coral reef** | | **Mangrove** | | **Seagrass bed** | | **Coral reef** | |
|  |  | n | [CHD] | δ^15^N | [CHD] | δ^15^N | [CHD] | δ^15^N | [CHD] | δ^15^N | [CHD] | δ^15^N | [CHD] | δ^15^N |
| *Oreaster reticulatus* | E | 3 |  |  |  |  |  |  |  |  | 253.0 ± 129.9 | 9.3 ± 0.2 |  |  |
| *Panulirus argus* | C | 9 |  |  | 102.0 ± 29.7 | 6.5 ± 0.1 | 86.7 ± 10.4 | 7.3 ± 0.1 |  |  |  |  | 86.7 ± 18.5 | 7.7 ± 0.4 |
| *Panulirus guttatus* | C | 5 |  |  |  |  | 144.0 ± 3.6 | 8.4 ± 0.6 |  |  |  |  | 68.0 | 8.4 |
| *Petrochirus diogenes* | C | 1 |  |  | 265.0 | 7.4 |  |  |  |  |  |  |  |  |
| *Stegastes planifrons* | T | 3 |  |  |  |  | 17.3 ± 8.7 | 8.6 ± 0.2 |  |  |  |  |  |  |
| *Farfantepenaeus subtilis* | C | 2 | 430.0 | 4.6 |  |  |  |  | 227.0 | 6.0 |  |  |  |  |
| *Xiphopenaeus kroyeri* | C | 1 |  |  |  |  |  |  | 189.0 | 5.3 |  |  |  |  |
| **Symbiotic organisms** |  |  |  |  |  |  |  |  |  |  |  |  |  |  |
| *Porites astreoides* | Cn | 5 |  |  |  |  | 1.6 | 3.5 |  |  |  |  | 2.4 | 4.2 ± 0.1 |
| *Porites furcata* | Cn | 9 |  |  | 11.3 ± 0.6 | 2.3 ± 1.0 | 2.6 ± 0.4 | 3.6 ± 0.4 |  |  |  |  | 1.9 ± 0.5 | 4.0 ± 0.1 |
| *Stichodactyla helianthus* | Cn | 6 |  |  |  |  | 41.7 ± 6.0 | 5.2 ± 0.1 |  |  |  |  | 11.5 ± 2.3 | 4.6 ± 0.0 |
| **Total** |  |  | **182.4 ± 22.6** | **6.7 ± 1.5** | **54.1 ± 8.9** | **5.1 ± 2.4** | **53.0 ± 8.2** | **6.5 ± 2.8** | **251.0 ± 52.7** | **6.9 ± 2.6** | **71.5 ± 10.6** | **7.0 ± 3.1** | **47.2 ± 6.0** | **7.0 ± 2.7** |
